# Supplementary material for: The Secretomes of Painful Versus Nonpainful Human Schwannomatosis Tumor Cells Differentially Influence Sensory Neuron Gene Expression and Sensitivity
Source: Sci Rep. 2019 Sep 11;9:13098. doi: 10.1038/s41598-019-49705-w (PMC6739480; doi:10.1038/s41598-019-49705-w)
Supplement: Supplementary file 1 — Supplementary figures [file 41598_2019_49705_MOESM1_ESM.pdf]

The Secretomes of Painful Versus Nonpainful Human Schwannomatosis Tumor Cells Differentially Influence Sensory Neuron Gene Expression and Sensitivity

Kimberly Laskie Ostrow[1,5*], Katelyn J. Donaldson[1]; Randy Rubright[1]; Michael J. Caterina [2,3,4,5]; Allan Belzberg[2,5] Ahmet Hoke[1]

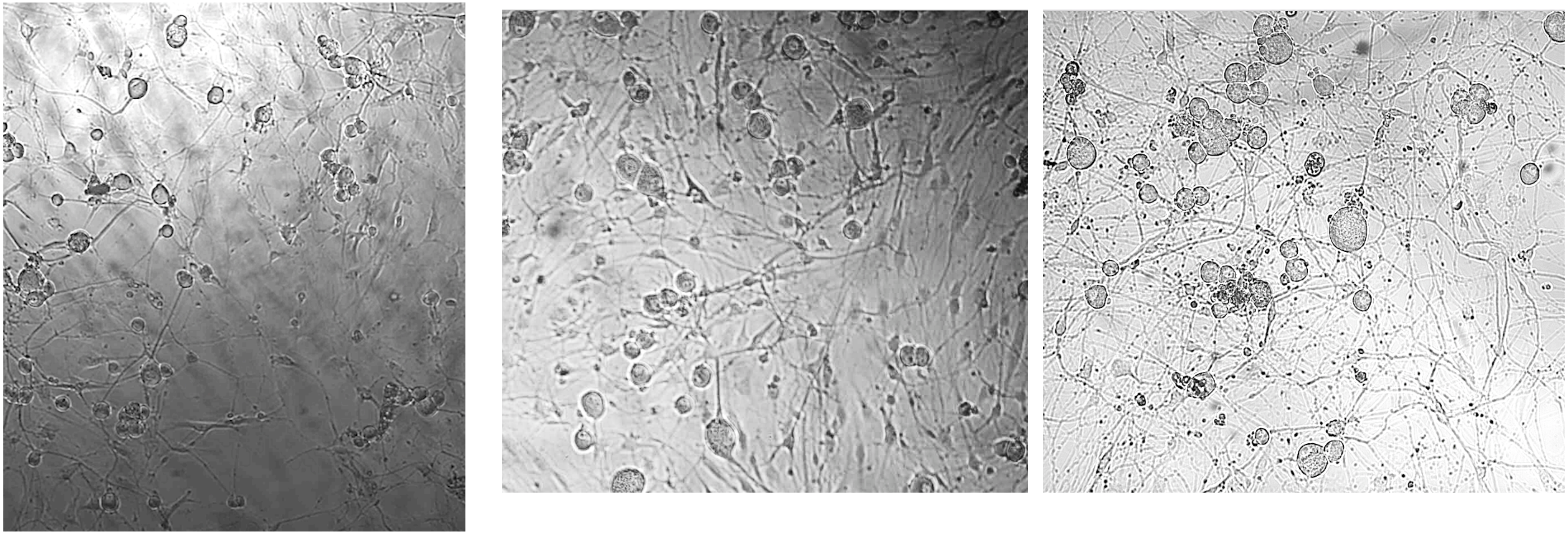

Painful Tumor 1 CM | Painful Tumor 2 CM | Non-Painful Tumor 2 CM

Supplemental Figure 1: Brightfield images of CM-treated DRGs prior to Calcium imaging. Neurite sprouting can be visualized in DRGs after 48 hours of incubation with SWN tumor CM regardless of pain status.

Dose response to KCl After treatment with NP Tumor CM

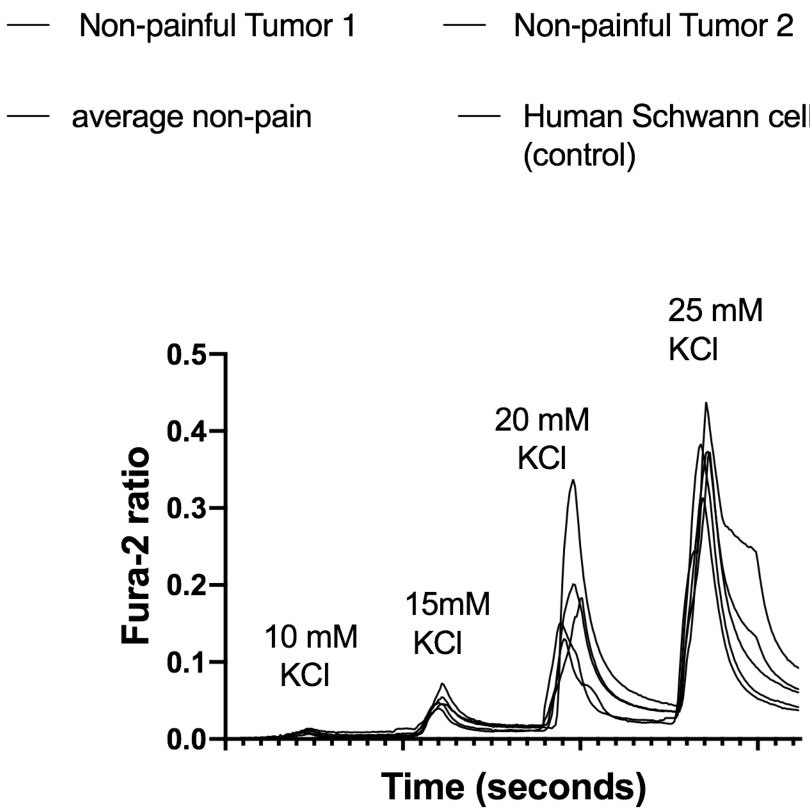

Supplemental Figure 2:
KCL dose response after pre-treatment with 3 separate non-painful tumor CMs. AUC was not statistically significant between groups.

KCL Dose	Average AUC NP Tumor 1	Average AUC NP Tumor 2	Average AUC NP Tumor 3	p value
10 mM	0.243 (+/- 0.07)	0.120 (+/- 0.08)	0.221 (+/- 0.06)	p=0.37
15 mM	0.946 (+/- 0.12)	0.85 (+/- 0.24)	1.14 (+/- 0.16)	p=0.4
20 mM	3.12 (+/- 0.51)	2.86 (+/- 0.53)	4.2 (+/- 0.59)	p=0.2
25 mM	6.45 (+/- 1.68)	6.92 (+/- 1.58)	6.94 (+/- 0.74)	p-0.9

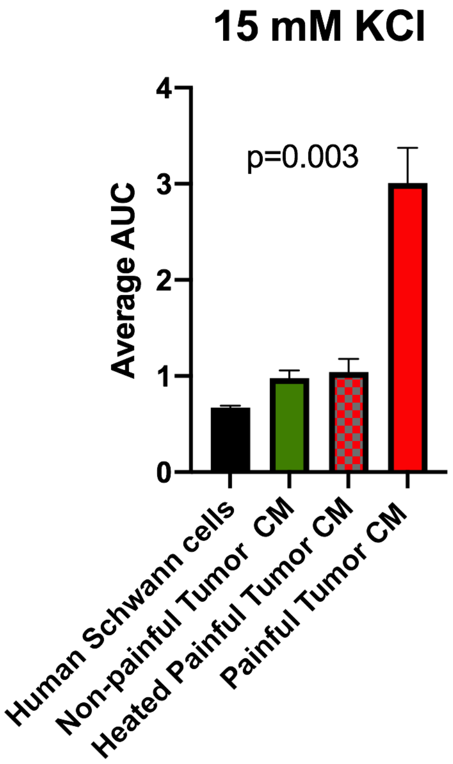

Supplemental Figure 3: Effect of Heating painful Tumor CM. Heating the painful CM at 80C for 30 min (**red patterned**) reduced responsiveness to that of non-painful CM (green)

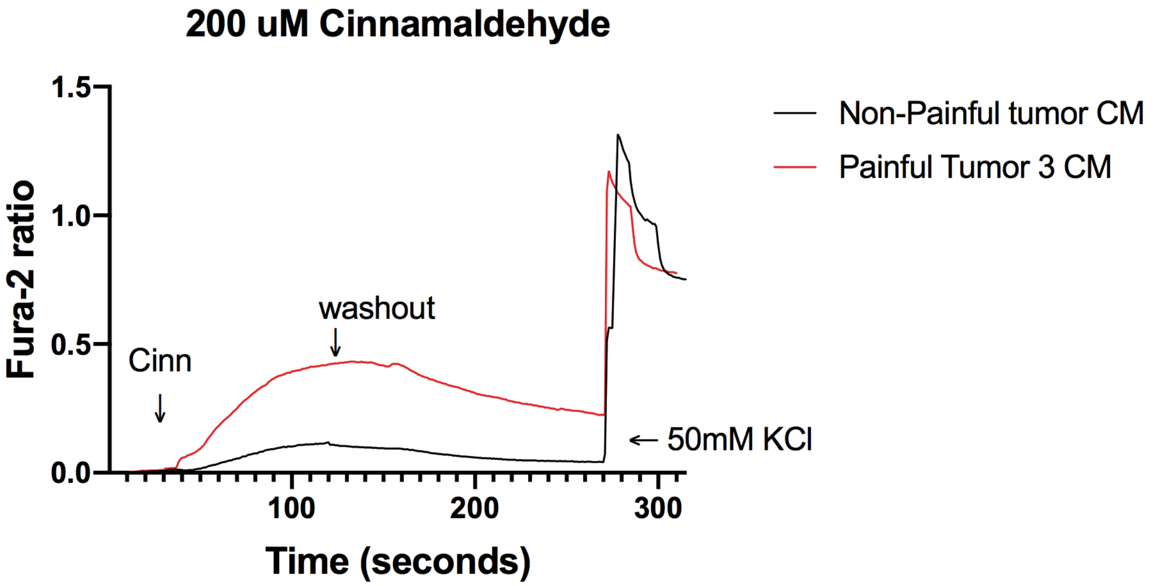

Supplemental Figure 4: DRG cells were perfused 200 uM Cinnamaldehyde for 120 sec with a washout with 3mM KCl buffer for and additional 2 minutes. Fura-2 ratio measurements were recorded at 2 second intervals. The datapoints were corrected for differing baseline readings of Fura-2. Area under the curve was calculated for each treatment. The effect of cinnamaldehyde started 30-60 seconds after treatment peaked at 120 seconds, and lasted for 4 minutes. Painful tumor 3 CM increased the responsiveness of the DRGs to cinnamaldehyde as calculated by area under the curve p=0.03.

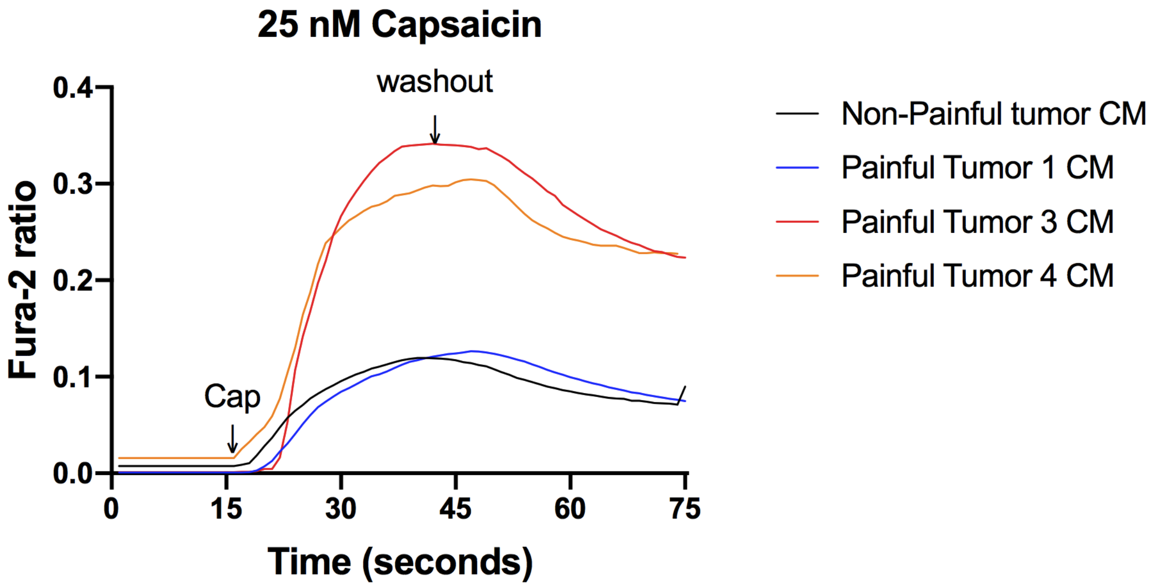

Supplemental Figure 5: DRG cells were perfused 25nM capsaicin for 30sec with a washout with 3mM KCl buffer for 2 minutes. Fura-2 ratio measurements were recorded at 2 second intervals. The datapoints were corrected for differing baseline readings of Fura-2. Area under the curve was calculated for each treatment. The effect of capsaicin started immediately peaked after 30 seconds lasted for 1 minute. CM from painful tumors 3 and 4 CM increased the responsiveness of the DRGs to capsaicin as calculated by area under the curve p=0.0001. One way ANOVA with multiple comparisons was employed to determine statistical significance.

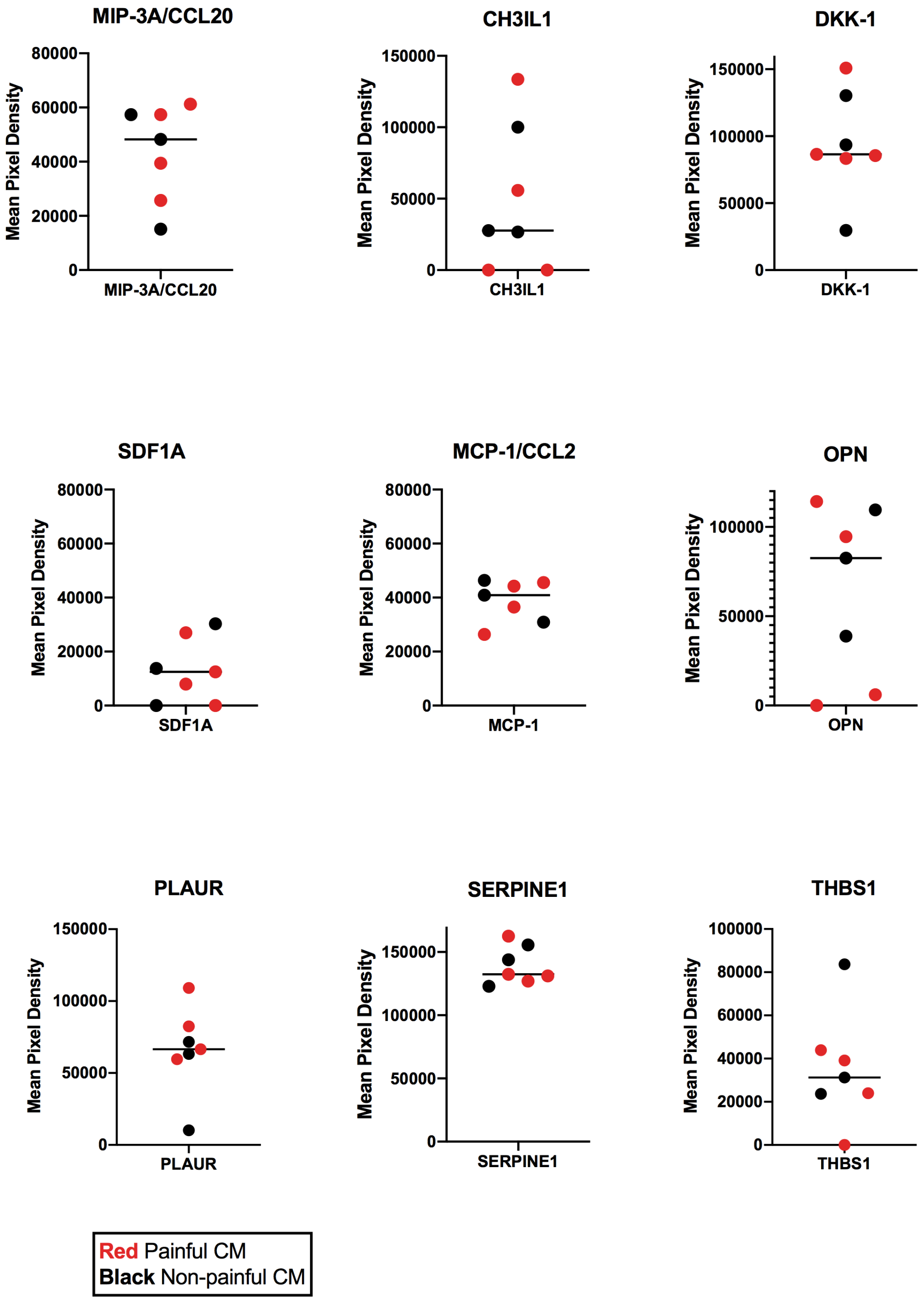

Supplemental Figure 6: Cytokines that were detected in CM that were similar between painful and Non-painful CM.

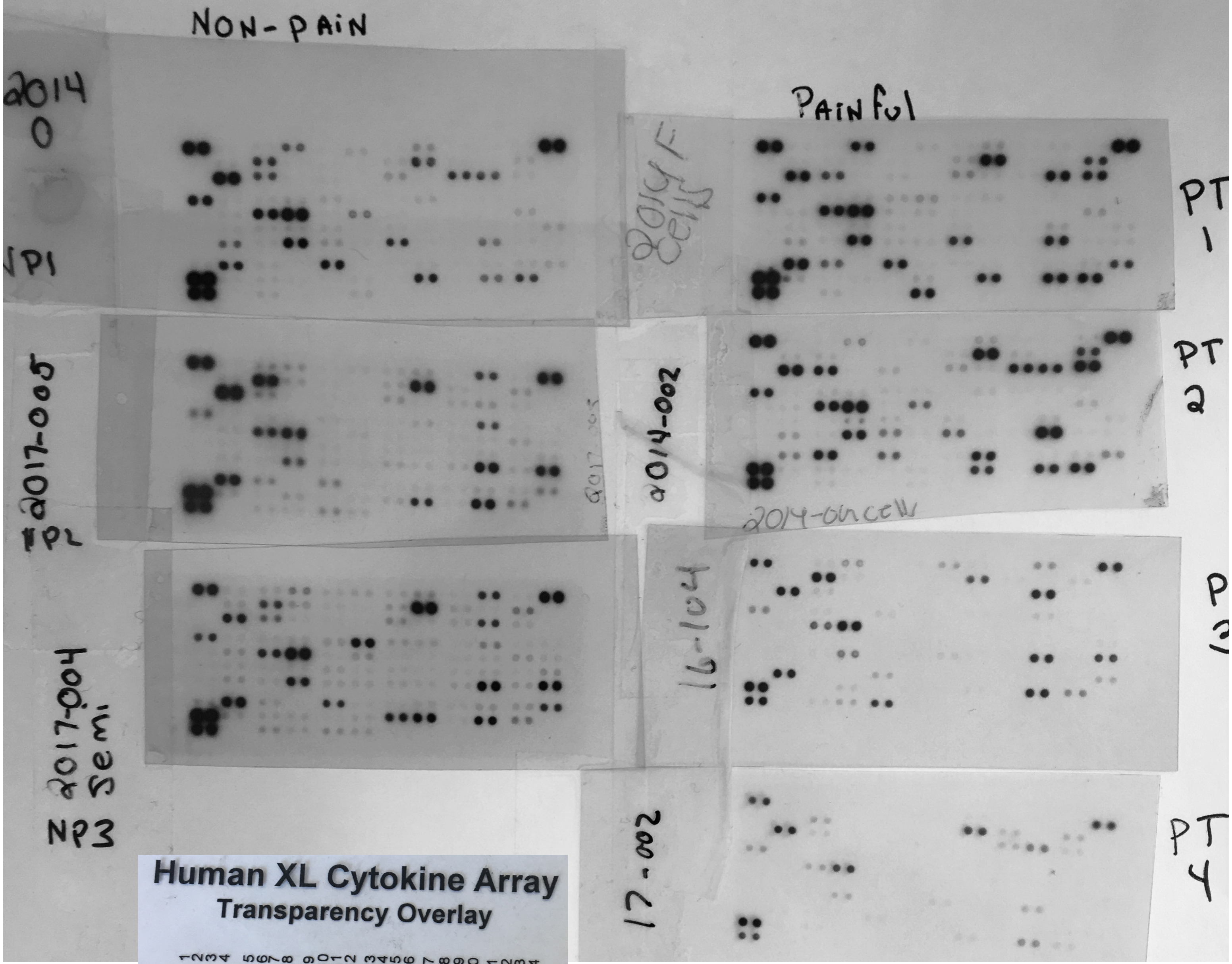

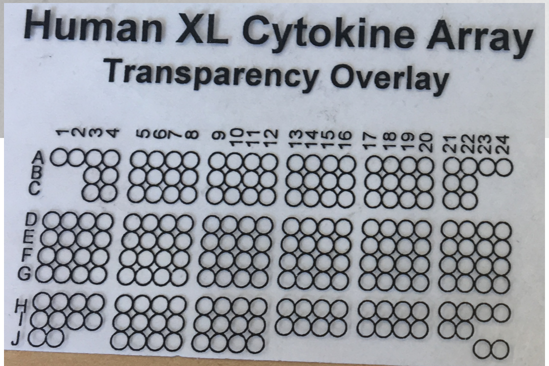

Supplemental Figure 7: Human cytokine array blots after 2 min exposure to X-ray film. Positions A 1,2 A23,24 and J 23, 24 are positive controls used for normalizing protein levels between blots.
